# Biomarker endpoints in cancer cachexia clinical trials: Systematic Review 5 of the cachexia endpoint series

**DOI:** 10.1002/jcsm.13491

**Published:** 2024-05-23

**Authors:** Michael S. Yule, Joshua Thompson, Khachonphat Leesahatsawat, Mariana S. Sousa, Stefan D. Anker, Jann Arends, Trude R. Balstad, Leo R. Brown, Asta Bye, Olav Dajani, Marie Fallon, Marianne J. Hjermstad, Gunnhild Jakobsen, James McDonald, Josh McGovern, Eric J. Roeland, Judith Sayers, Richard J.E. Skipworth, Inger O. Ottestad, Iain Philips, Melanie R. Simpson, Tora S. Solheim, Ola Magne Vagnildhaug, Donald McMillan, Barry J.A. Laird, Ross D. Dolan, the Cancer Cachexia Endpoints Working Group

**Affiliations:** ^1^ St Columba's Hospice Edinburgh UK; ^2^ Edinburgh Cancer Research Centre University of Edinburgh Edinburgh UK; ^3^ Academic Department of Surgery University of Glasgow, New Lister Building, Glasgow Royal Infirmary Glasgow UK; ^4^ Improving Palliative, Aged and Chronic Care through Clinical Research and Translation (IMPACCT) University of Technology Sydney Sydney Australia; ^5^ Department of Cardiology (CVK) Berlin Institute of Health Center for Regenerative Therapies (BCRT) Berlin Germany; ^6^ Institute of Heart Diseases Wroclaw Medical University Wroclaw Poland; ^7^ German Centre for Cardiovascular Research (DZHK) partner site Berlin, Charité Universitätsmedizin Berlin Berlin Germany; ^8^ Department of Medicine I Medical Center – University of Freiburg, Faculty of Medicine, University of Freiburg Freiburg im Breisgau Germany; ^9^ Department of Clinical Medicine, Clinical Nutrition Research Group UiT The Arctic University of Norway Tromsø Norway; ^10^ Department of Clinical and Molecular Medicine, Faculty of Medicine and Health Sciences Norwegian University of Science and Technology Trondheim Norway; ^11^ Department of Clinical Surgery University of Edinburgh, Royal Infirmary of Edinburgh Edinburgh UK; ^12^ Regional Advisory Unit for Palliative Care, Department of Oncology Oslo University Hospital, University of Oslo Oslo Norway; ^13^ European Palliative Care Research Centre, Department of Oncology Oslo University Hospital and Institute of Clinical Medicine, University of Oslo Oslo Norway; ^14^ Department of Nursing and Health Promotion, Faculty of Health Sciences OsloMet‐Oslo Metropolitan University Oslo Norway; ^15^ Cancer Clinic St Olavs Hospital, Trondheim University Hospital Trondheim Norway; ^16^ Department of Public Health and Nursing, Faculty of Medicine and Health Sciences Norwegian University of Science and Technology Trondheim Norway; ^17^ Orgeon Health and Science University Portland OR USA; ^18^ Department of Nutrition, Institute of Basic Medical Sciences, Faculty of Medicine University of Oslo Oslo Norway; ^19^ The Clinical Nutrition Outpatient Clinic, Section of Clinical Nutrition, Department of Clinical Service, Division of Cancer Medicine Oslo University Hospital Oslo Norway

**Keywords:** biomarkers, cachexia, cancer, endpoints, trials

## Abstract

Regulatory agencies require evidence that endpoints correlate with clinical benefit before they can be used to approve drugs. Biomarkers are often considered surrogate endpoints. In cancer cachexia trials, the measurement of biomarkers features frequently. The aim of this systematic review was to assess the frequency and diversity of biomarker endpoints in cancer cachexia trials. A comprehensive electronic literature search of MEDLINE, Embase and Cochrane (1990–2023) was completed. Eligible trials met the following criteria: adults (≥18 years), prospective design, more than 40 participants, use of a cachexia intervention for more than 14 days and use of a biomarker(s) as an endpoint. Biomarkers were defined as any objective measure that was assayed from a body fluid, including scoring systems based on these assays. Routine haematology and biochemistry to monitor intervention toxicity were not considered. Data extraction was performed using Covidence, and reporting followed PRISMA guidance (PROSPERO: CRD42022276710). A total of 5975 studies were assessed, of which 52 trials (total participants = 6522) included biomarkers as endpoints. Most studies (*n* = 29, 55.7%) included a variety of cancer types. Pharmacological interventions (*n* = 27, 51.9%) were most evaluated, followed by nutritional interventions (*n* = 20, 38.4%). Ninety‐nine different biomarkers were used across the trials, and of these, 96 were assayed from blood. Albumin (*n* = 29, 55.8%) was assessed most often, followed by C‐reactive protein (*n* = 22, 42.3%), interleukin‐6 (*n* = 16, 30.8%) and tumour necrosis factor‐α (*n* = 14, 26.9%), the latter being the only biomarker that was used to guide sample size calculations. Biomarkers were explicitly listed as a primary outcome in six trials. In total, 12 biomarkers (12.1% of 99) were used in six trials or more. Insulin‐like growth factor binding protein 3 (IGFBP‐3) and insulin‐like growth factor 1 (IGF‐1) levels both increased significantly in all three trials in which they were both used. This corresponded with a primary outcome, lean body mass, and was related to the pharmacological mechanism. Biomarkers were predominately used as exploratory rather than primary endpoints. The most commonly used biomarker, albumin, was limited by its lack of responsiveness to nutritional intervention. For a biomarker to be responsive to change, it must be related to the mechanism of action of the intervention and/or the underlying cachexia process that is modified by the intervention, as seen with IGFBP‐3, IGF‐1 and anamorelin. To reach regulatory approval as an endpoint, the relationship between the biomarker and clinical benefit must be clarified.

## Introduction

Cancer cachexia is a devastating syndrome characterized by inflammation, anorexia and involuntary loss of muscle/fat.^1^, ^2^, ^3^, ^4^ Treatment options remain limited, and this is in part due to sub‐optimal clinical trial design, including a lack of clarity on the optimal endpoints to use. Endpoints in cancer cachexia trials can be split into various categories,^5^ often aligned with the various cachexia definitions.^3^ Yet using different endpoints simultaneously in trials can lead to conflicting results. To illustrate, the ROMANA trials^6^ found that anamorelin increased lean body mass but did not improve hand grip strength. There are multiple reasons why this may have happened, including endpoint sensitivity, the mechanism of action of the trial drug, temporal aspects or perhaps mainly population selection. A post hoc pooled analysis of these trials showed that participants who had systemic inflammation (measured using C‐reactive protein [CRP] and albumin, combined in the modified Glasgow prognostic score [mGPS]) had a better response to anamorelin, both considering lean body mass and hand grip strength, than those who did not.^7^ Recently, trials examining ponsegromab,^8^ which targets growth differentiation factor 15 (GDF‐15), have used elevated levels of GDF‐15 as both an entry criteria and an exploratory endpoint. These two examples provide some rationale for the role of biomarkers in cancer cachexia clinical trials; however, these are the exception rather than the rule. Further work in this area is required to help facilitate future trials.

The US Food and Drug Administration (FDA) states that endpoints can be either a clinical outcome, known as a direct measure (such as overall survival), or, in recent years, a surrogate endpoint.^9^ A biomarker is an example of a surrogate endpoint and is defined as an objective indication of the current medical state of an organism.^10^ For a surrogate endpoint to lead to the approval of a new drug, the endpoint must have extensive evidence to support its correlation with clinical benefit,^9^ and the biomarker must be reliably assessable, predictive and responsive.^11^ The FDA has approved several biomarkers to study the outcome of pharmacological interventions, such as serum insulin‐like growth factor 1 (IGF‐1) levels in patients with acromegaly.^12^ However, to date, no biomarker assayed from blood or body fluid has been featured as the primary endpoint for cancer drug approval, but they have formed part of a composite endpoint.^13^ In cancer cachexia, the picture is still less clear as an appraisal of endpoints has not been undertaken. Furthermore, it could be argued that there is less of a published, demonstrable relationship between cachexia biomarkers and clinical benefit.

The aim of this systematic review is to explore which biomarkers have been used in cancer cachexia trials and with what frequency and diversity.

## Methods

This systematic review is reported in accordance with the Preferred Reporting for Systematic Reviews and Meta‐Analyses (PRISMA) statement.^14^ The review protocol was prospectively registered at the International Prospective Register of Systematic Reviews: PROSPERO (CRD42022276710).^15^ This systematic review is part of a collaboration reviewing different endpoints in cancer cachexia trials.^16^, ^17^

### Search strategy

A systematic search of the MEDLINE (OVID), Embase (OVID) and Cochrane Central Register of Controlled Trials databases was conducted by a senior research librarian (University of Oslo), and studies from 1 January 1990 to 17 October 2023 were assessed. Search results were synthesized and managed using the web‐based systematic review software ‘Covidence’ (Veritas Health Innovations, Melbourne, Australia), and duplicates were removed. A detailed search strategy is outlined in *Appendix* *S1*.

### Study eligibility criteria

Prospective comparative clinical trials that considered an intervention aiming to treat or attenuate the effects of cachexia in adult patients (≥18 years) with cancer were considered for eligibility. Analysis of a biomarker (defined as an objective measure assayed from body fluid measured at baseline and at the end of a trial) was used as an inclusion criterion. Biomarkers used to evaluate only baseline characteristics were not included. All routine haematology and biochemistry tests that were explicitly stated as being used to measure intervention toxicity/safety were not included. In addition, routine haematology and biochemistry tests stated to have been obtained from the participant in the study methodology but not reported were also not included. Biomarkers used to measure the compliance of an intervention were considered separately. Inclusion was irrespective of the site of primary malignancy, modality of intervention (e.g., pharmacological, nutritional and physical exercise) or choice of comparator. Trials were excluded if they studied fewer than 40 patients and/or if the intervention lasted <14 days. All included full‐text articles were written in the English language.

### Data selection and extraction

The titles and abstracts of the identified studies were independently reviewed by three authors (O. D., T. S. S. and B. L.). Those selected were subsequently subject to full text review (R. D., M. S., M. Y. and J. T.). A pre‐defined data extraction table was developed (R. D., M. S. and M. Y.) and pilot‐tested before relevant data points were extracted (M. Y.).

### Assessing risk of bias

The methodological quality of each study was independently assessed by four reviewers (J. S., J. M., O. D. and B. L.) using the modified Downs and Black checklist.^18^ This tool assesses several criteria, including study design, internal and external validity and responding standards. A total score of 28 is possible for randomized trials and 25 for non‐randomized studies. Previous investigators^19^ have classified scores as excellent (26–28), good (20–25), fair (15–19) and poor (<15).

### Data analysis

In assessing the frequency and diversity of biomarkers used in cachexia intervention trials, study characteristics, participant details and disease demographics are reported descriptively. Additionally, the large number and heterogeneous nature of the trials included meant that a meta‐analysis of treatment effects on each endpoint was not feasible. Visualizations were conducted using RStudio Version 4.2.2 (R Foundation for Statistical Computing, Vienna, Austria), with packages including tidyverse.

## Results

**Figure 1:**
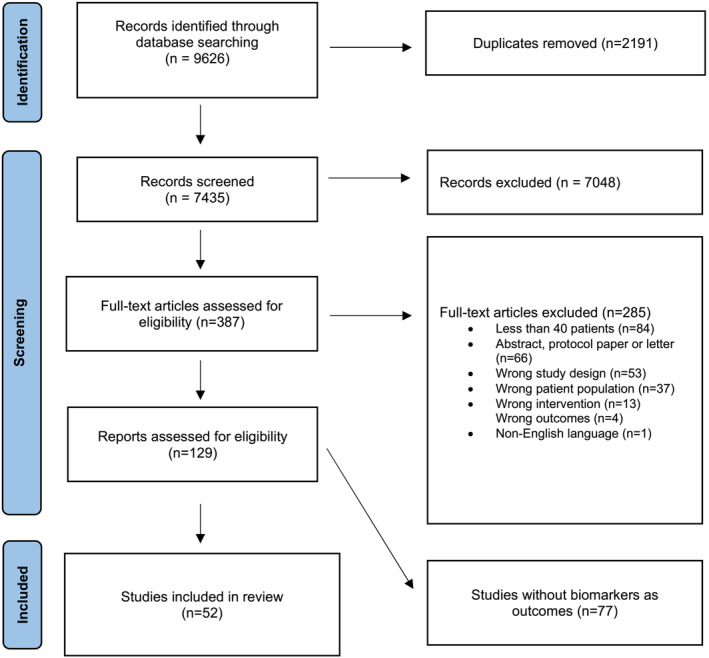
PRISMA diagram.

*Figure* *1* details the PRISMA diagram. After the removal of duplicates, 7435 records were reviewed by title or abstract (the abstract was assessed where the title was insufficient), resulting in 387 records being appraised in full. Following appraisal, 285 records were further excluded, leaving 129 that were eligible for the systematic review database. Of these, 52 studies analysed biomarker endpoints and thus were eligible to be included (*Figure* *1*).

*Table* *1* shows the key characteristics of eligible trials. There were 52 trials (total participants = 6522), of which 49 (94.2%) were randomized control trials. There was one dose escalation study^44^ and two^56^, ^60^ non‐randomized trials. Most studies (*n* = 29, 55.7%) included multiple cancer types; however, in trials in which a single cancer was examined, this was commonly lung (*n* = 5, 9.6%), head and neck (*n* = 5, 9.6%), gastrointestinal (*n* = 5, 9.6%) or pancreatic (*n* = 4, 7.7%). Intention to treat numbers were recorded as sample size, and these ranged from 40 participants to 518. Eight trials were international. Single‐country trials included Italy (*n* = 5, 9.6%), China (*n* = 5, 9.6%), Germany (*n* = 4, 7.7%) and Japan (*n* = 4, 7.7%). Pharmacological interventions (*n* = 27, 51.9%) were most commonly evaluated, followed by nutritional (*n* = 20, 38.4%) and multimodal (*n* = 6, 11.5%) interventions. Exercise and lifestyle interventions were not used in isolation in any of the included trials.

**Table 1:** Key characteristics of the included trials

First author (reference)	Year	Sample size	Study design	Study quality	Primary cancer site	Intervention	Comparator	Biomarker	Primary outcome
Chen^20^	2023	108	RCT	8	Gastrointestinal	Intensive, individualized nutritional counselling, education + nutritional supplements **(Nutritional)**	Nutritional counselling	**Albumin, Hb, pre‐albumin** , Cr, lymphocytes, Plts, total protein, WBC, neutrophils, **urea**	**Nutritional status**
Kutz^21^	2022	69	RCT	7	Head and neck	Nutritional counselling **(Nutritional)**	Standard care	CRP	QoL, nutritional status
Sim^22^	2022	58	RCT	8	Gastrointestinal	Omega 3‐enriched oral nutritional supplement + nutritional counselling **(Nutritional)**	Nutritional counselling	Albumin, IL‐6, TNF‐α, Hb, pre‐albumin, transferrin, cholesterol, IL‐8	Not explicitly stated
Ko^23^	2021	40	Pilot RCT	8	Any solid malignancy	Yukgunja‐tang **(Nutritional)**	Nutritional counselling	IL‐6, TNF‐α, ghrelin	**Anorexia–Cachexia Subscale (FAACT)**
Balstad^24^	2021	46	Phase II RCT	8	NSCLC + pancreatic	Exercise + NSAID + ONS containing polyunsaturated fatty acids **(Multimodal)**	Standard care	CRP, IGF‐1, **EPA, DHA** , adiponectin, **DPA** , glycerol, lipolytic activity, **vitamin D** , ZAG	Compliance, recruitment
Hunter^25^	2021	120	Phase III RCT	7	Any solid tumour	Mirtazapine **(Pharmacological)**	Placebo	CRP, IL‐6, YKL‐40	Appetite
Izumi^26^	2021	81	RCT	6	Any malignancy (+ late‐onset hypogonadism)	Testosterone enanthate **(Pharmacological)**	Standard care	IL‐6, **TNF‐α** , Hb, IGF‐1, PSA, free testosterone, FSH, LH, total testosterone	Not explicitly stated
Movahed^27^	2020	100	RCT	8	Oesophageal	Supplements ± enteral or parenteral nutrition ± pharmacotherapy + nutritional counselling **(Multimodal)**	Nutritional counselling	Albumin, CRP, Hb, Cr, lymphocytes, Plts, total protein, ALT, Hct, triglyceride, WBC, AST, HDL, LDL, **urea** , cholesterol, MCV, MCH, RDW	**PG‐SGA**
Qiu^28^	2020	96	RCT	6	Oesophageal	Nutritional counselling **(Nutritional)**	Standard care	**Albumin, total protein**	**Complications of chemotherapy** , efficacy
Huang^29^	2020	114	RCT	7	Nasopharyngeal	Oral nutritional supplement **(Nutritional)**	Standard care	**Albumin** , pre‐albumin, **total protein**	Body weight
Laviano^30^	2019	56	Pilot RCT	8	NSCLC	Targeted medical nutrition supplement **(Nutritional)**	Isocaloric comparator drink	Albumin, CRP, IL‐6, TNF‐α, triglyceride, glucose, total cholesterol, HbA1c, HDL, LDL, IL‐8, insulin, IL‐15, NLR^a^	Adverse events
Akita^31^	2019	62	RCT	8	Pancreatic	EPA‐enriched nutritional supplement **(Nutritional)**	Standard care	Albumin, pre‐albumin, lymphocytes, triglyceride, total cholesterol, HbA1c	Skeletal muscle mass, psoas muscle area
Katakami^32^	2018	174	Phase III RCT	8	NSCLC	Anamorelin **(Pharmacological)**	Placebo	Pre‐albumin, **IGF‐1, IGFBP‐3**	**Lean body mass**
Kouchaki^33^	2018	90	Phase III RCT	8	Gastrointestinal	Megestrol acetate + celecoxib **(Pharmacological)**	Megestrol acetate + placebo	Albumin, CRP, IL‐6, GPS	Body weight
Bumrungpert^34^	2018	48	RCT	6	Any malignancy	40 g of whey protein isolates + zinc + selenium **(Nutritional)**	Maltodextrin oral snack	**Albumin** , CRP, Hb, Cr, Plts, ALT, Hct, WBC, AST, urea, ALP, CD4+, **GSH, IgG** , RBC	Not explicitly stated
Xie^35^	2018	54	RCT	8	Lung	Thalidomide + cinobufagin **(Pharmacological)**	Cinobufagin	Albumin ^a^	MUAC, **weight, EORTC QLQ‐C30** , side effects, **grip strength, overall survival**
Schink^36^	2018	131	Pilot RCT	9	Any solid tumour	Whole‐body electromyostimulation + nutritional counselling **(Multimodal)**	Nutritional counselling	Albumin, CRP, Hb, Cr, leucocytes, total protein, Hct, thrombocytes	**Skeletal muscle mass**
Turcott^37^	2018	47	RCT	7	NSCLC	Nabilone **(Pharmacological)**	Placebo	Albumin, Hb, lymphocytes, Plts, leucocytes, neutrophils, NLR, PLR	Not explicitly stated
Solheim^38^	2017	46	Phase II RCT	8	NSCLC + pancreatic	Exercise, celecoxib + nutritional supplements **(Multimodal)**	Standard care	CRP	Feasibility, **compliance**
Zietarska^39^	2017	95	RCT	6	Colorectal	Nutritional supplements **(Nutritional)**	Standard care	**Albumin** , CRP, Hb, **pre‐albumin** , Plts, triglycerides, WBC, total cholesterol, **ferritin** , neutrophils	Toxicity
Werner^40^	2017	60	RCT	7	Pancreatic	Fish oil **(Nutritional)**	Marine phospholipids	Albumin, CRP, leucocytes, ALT, triglycerides, EPA, thrombocytes, total cholesterol, DHA, HDL, LDL, arachidonic acid, cholinesterase, AST, LDL/HDL ratio, phospholipids, VLDL	Body weight, appetite
Takayama^41^	2016	181	Phase II RCT	8	NSCLC	Anamorelin 100 mg **OR** anamorelin 50 mg **(Pharmacological)**	Placebo	IL‐6, TNF‐α, **pre‐albumin, IGF‐1, IGFBP‐3** , acyl‐ghrelin, desacyl‐ghrelin, IL‐1β	**Lean body mass** , hand grip strength
Gavazzi^42^	2016	79	RCT	7	Upper gastrointestinal	Continue enteral nutrition via jejunostomy **(Nutritional)**	Nutritional counselling	Albumin, pre‐albumin, lymphocytes, transferrin, cholinesterase	**Body weight**
Garcia^43^	2015	82	Phase II RCT	7	Any malignancy	Anamorelin 50 mg **(Pharmacological)**	Placebo	CRP, IL‐6, TNF‐α, **IGF‐1, glucose, IGFBP‐3** , insulin	**Lean body mass**
Hong^44^	2014	52	Dose escalation	8	Multiple	MABp1 (anti‐IL‐1α antibody) **(Pharmacological)**	Dose escalation study	CRP, IL‐6, Plts, CD14 expression, CD16 expression, IL‐1α expression	Toxicity
Poulsen^45^	2014	61	RCT	5	Oesophageal + gastric + gynaecological	Nutritional counselling **(Nutritional)**	Standard care	Albumin, Hb, transferrin, Fe, ferritin, cobalamin, magnesium, phosphate	Body weight
Pottel^46^	2014	85	RCT	8	Head and neck	Echium oil **(Nutritional)**	Sunflower oil	**n‐3 EPA** , n‐6 AA, **n‐3 ALA, n‐6 DGLA, n‐6 GLA, n‐6 LA**	Body weight
Dobs^47^	2013	159	Phase II RCT	8	Other mixed	Enobosarm 1 mg **OR** enobosarm 3 mg **(Pharmacological)**	Placebo	CRP, IL‐6, TNF‐α, HbA1c, PSA, bone‐specific ALP, C‐telopeptide, N telopeptide, osteocalcin	**Lean body mass**
Kanat^48^	2013	69	RCT	8	Any malignancy	Megestrol acetate + meloxicam **OR** megestrol acetate + EPA‐enriched nutritional supplement **(Multimodal)**	Meloxicam + EPA‐enriched nutritional supplement	IL‐6, TNF‐α	**Body weight, lean body mass**
Del Fabbro^49^	2013	73	RCT	10	Gastrointestinal + lung	Melatonin **(Pharmacological)**	Placebo	CRP	Appetite
Maccio^50^	2012	144	Phase III RCT	8	Gynaecological	Megestrol acetate + l‐carnitine + celecoxib + antioxidants **(Pharmacological)**	Megestrol acetate	CRP, **IL‐6, TNF‐α** , GPS, glutathione peroxidase, **reactive oxygen species, leptin** , superoxide dismutase	**Lean body mass, resting energy expenditure, fatigue, EORTC QLQ‐C30**
Kraft^51^	2012	72	RCT	10	Pancreatic	l‐Carnitine supplement **(Nutritional)**	Placebo	Albumin, TNF‐α^a^, leucocytes, CA19‐9	Biomarker only
Wen^52^	2012	108	RCT	5	Any malignancy	Megestrol acetate + thalidomide **(Pharmacological)**	Megestrol acetate	**IL‐6, TNF‐α, GPS**	**Body weight, fatigue, EORTC QLQ‐C30**
Madeddu^53^	2012	60	Phase III RCT	7	Any malignancy	l‐Carnitine + celecoxib + megestrol acetate **(Pharmacological)**	l‐Carnitine + celecoxib	CRP, IL‐6, TNF‐α, GPS	Lean body mass
Mantovani^54^	2010	332	Phase III RCT	7	Any malignancy	Megestrol acetate **OR** EPA‐enriched nutritional supplement **OR** l‐carnitine **OR** thalidomide **(Pharmacological)**	Megestrol acetate + EPA‐enriched nutritional supplement + l‐carnitine + thalidomide	IL‐6, TNF‐α, GPS, glutathione peroxidase, reactive oxygen species	**Lean body mass, resting energy expenditure**
Fearon^55^	2006	518	RCT	8	Gastrointestinal + lung	EPA 2 g **OR** EPA 4 g **(Nutritional)**	Placebo	Albumin, CRP	Body weight, lean body mass
Gonçalves Dias^56^	2005	64	Non‐randomized trial	1	Head and neck	Home enteral (nasogastric) feeding **OR** oral diet + nutritional supplement **(Nutritional)**	Oral diet	Albumin, Hb, lymphocytes, total proteins, Hct	**Caloric and protein ingestion**
Lundholm^57^	2004	309	RCT	5	Any solid tumour	Indomethacin + erythropoietin + nutritional support + home total parenteral nutrition **(Multimodal)**	Indomethacin + erythropoietin	**Albumin** , CRP, Hb, Cr, IGF‐1, ALT, WBC, AST, thrombocytes, Fe, **ALP** , bilirubin, ESR, free T3	Body composition, resting energy expenditure, maximum exercise capacity
Bruera^58^	2003	91	RCT	7	Any malignancy	Fish oil capsules **(Nutritional)**	Placebo	EPA, DHA	Appetite
Jatoi^59^	2002	469	RCT	8	Any malignancy (not brain, breast, ovarian or endometrial)	Megestrol acetate + dronabinol **OR** megestrol acetate + placebo **(Pharmacological)**	Dronabinol + placebo	IL‐6^a^	Biomarker only
Barber^60^	1999	42	Non‐randomized trial	2	Pancreatic	Supplement with 2.18 g of EPA + 0.92 g of docosahexaenoic acid **OR** supportive care **(Nutritional)**	Standard care	**Albumin** ^**a**^ **, CRP** ^**a**^ **, pre‐albumin** ^**a**^ **, transferrin** ^**a**^, ceruloplasmin^a^, haptoglobin^a^, α‐1 antitrypsin^a^, α‐1‐acid glycoprotein^a^	Biomarker only
McMillan^61^	1999	73	RCT	7	Gastrointestinal	Megestrol acetate + ibuprofen **(Pharmacological)**	Megestrol acetate + placebo	Albumin, CRP	**Body weight, skinfold thickness** , appetite
Daneryd^62^	1998	108	RCT	7	Any malignancy	Indomethacin + erythropoietin (Pharmacological)	Indomethacin	**Albumin, CRP, leucocytes, MCHC** , thrombocytes, **Fe, ESR, free T3** , MCV, urinary nitrogen, reticulocytes, TIBC, urinary Cr	Not explicitly stated
Vadell^63^	1998	150	RCT	7	Any malignancy	Megestrol acetate 480 mg **OR** megestrol acetate 160 mg **(Pharmacological)**	Placebo	Albumin, pre‐albumin, ferritin	Not explicitly stated
Chen^64^	1997	129	RCT	8	Head and neck	Megestrol acetate **OR** prepulside **(Pharmacological)**	Placebo	Albumin	**Body weight, appetite**
Beller^65^	1997	240	RCT	4	Any malignancy (not hormone dependent)	Megestrol acetate 480 mg **OR** megestrol acetate 160 mg **(Pharmacological)**	Placebo	Albumin, Hb, pre‐albumin, Cr, lymphocytes, folate	**QoL** , nutritional status
Neri^66^	1997	279	RCT	4	Any solid tumour	1000 mg of medroxyprogesterone acetate **(Pharmacological)**	Standard care	**Glucose**	Therapeutic impact questionnaire
Lissoni^67^	1996	100	RCT	7	Any solid tumour	Melatonin **(Pharmacological)**	Standard care	TNF‐α^a^	**Body weight**
Loprinzi^68^	1993	351	Phase III RCT	8	Any malignancy other than brain	Megestrol acetate 1280 mg **OR** megestrol acetate 800 mg **(Pharmacological)**	Megestrol acetate 480 mg **OR** megestrol acetate 160 mg	Albumin	Not explicitly stated
Ovesen^69^	1993	137	RCT	8	SCLC + ovarian + breast	Nutritional counselling **(Nutritional)**	Standard care	Albumin	Body weight, quality of life
Downer^70^	1993	60	RCT	1	Any malignancy	Medroxyprogesterone acetate **(Pharmacological)**	Placebo	Albumin, transferrin, urinary nitrogen, retinol binding protein, thyroxine binding pre‐albumin	Not explicitly stated
Feliu^71^	1992	150	RCT	5	Any malignancy (not hormone dependent)	Megestrol acetate 240 mg **(Pharmacological)**	Placebo	Hb, Cr, Plts, leucocytes, ALT, AST, glucose, bilirubin	Body weight, **subject sense of appetite score** , Karnofsky performance status

*Note*: Sample sizes are reported as per ‘intention to treat’. Outcomes that are bold underlined had a statistically significant difference between groups.

Abbreviations: AA, arachidonic acid; ALA, alpha lipoic acid; ALP, alkaline phosphatase; ALT, alanine transaminase; AST, aspartate transaminase; CA19‐9, carbohydrate antigen 19‐9; Cr, creatinine; CRP, C‐reactive protein; DGLA, dihomo‐gamma‐linolenic acid; DHA, docosahexaenoic acid; DPA, docosapentaenoic acid; EORTC QLQ‐C30, European Organisation for Research and Treatment of Cancer Quality of Life Questionnaire Core 30; EPA, eicosapentaenoic acid; ESR, erythrocyte sedimentation rate; FAACT, Functional Assessment of Anorexia/Cachexia Therapy; Fe, iron; FSH, follicular stimulating hormone; GLA, gamma‐linolenic acid; GPS, Glasgow prognostic score; GSH, glutathione; Hb, haemoglobin; Hct, haematocrit; HDL, high‐density lipoprotein; IGF‐1, insulin‐like growth factor 1; IGFBP‐3, insulin‐like growth factor binding protein 3; IgG, immunoglobulin G; IL, interleukin; LA, linoleic acid; LDL, low‐density lipoprotein; LH, luteinizing hormone; MABp1, mammalian actin binding protein 1; MCH, mean corpuscular haemoglobin; MCHC, mean corpuscular haemoglobin concentration; MCV, mean corpuscular volume; MUAC, mid‐upper arm circumference; NLR, neutrophil‐to‐lymphocyte ratio; NSAID, nonsteroidal anti‐inflammatory drug; NSCLC, non‐small cell lung cancer; ONS, oral nutritional supplement; PG‐SGA, Patient‐Generated Subjective Global Assessment; PLR, platelet‐to‐lymphocyte ratio; Plts, platelets; PSA, prostate‐specific antigen; QoL, quality of life; RBC, red blood cell count; RCT, randomized controlled trial; RDW, red cell distribution width; SCLC, small cell lung cancer; TIBC, total iron binding capacity; TNF, tumour necrosis factor; VLDL, very low‐density lipoprotein; WBC, white blood cell count; ZAG, zinc‐α2‐glycoprotein.

^a^ Listed as a primary outcome or used in the power calculation implying a primary outcome.

In total, 99 different biomarkers were used across the 52 different trials (see *Appendix* *S2*). Ninety‐seven of these biomarkers were assayed from blood. Two (nitrogen and creatinine) were assayed from urine in the same trial, dated 1998.^62^ Biomarkers were explicitly listed as a primary outcome in six trials (11.5%). Two trials^26^, ^53^ used a biomarker as part of their entry criteria, and one trial was powered for a biomarker, tumour necrosis factor‐α (TNF‐α).^51^ Only one tumour‐specific biomarker, prostate‐specific antigen (PSA), was measured across the included trials. It was used in two trials^26^, ^47^ and statistically did not change significantly in either. Overall, most biomarkers (*n* = 53, 53.5%) were used only in one clinical trial.

**Figure 2:**
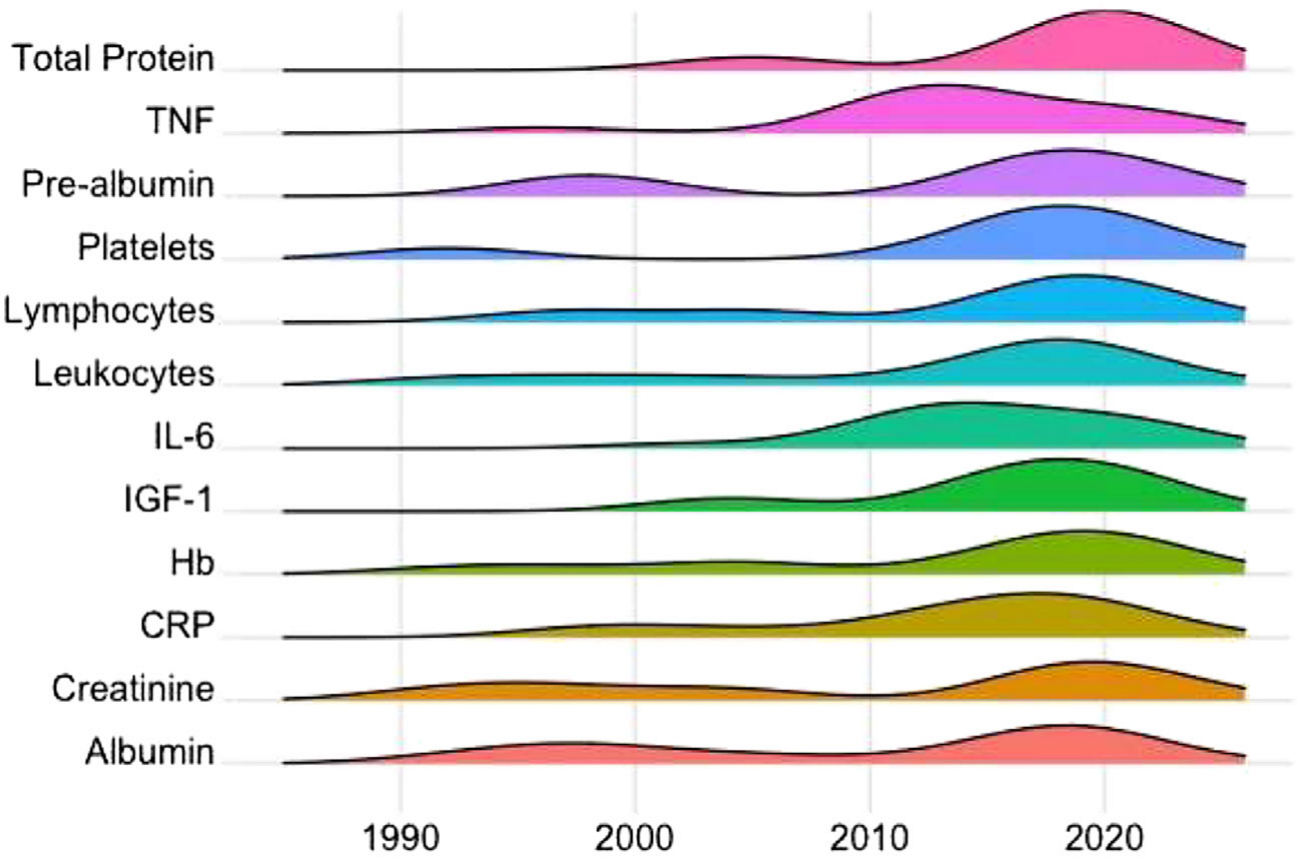
Temporal trends in the relative use of biomarkers in cancer cachexia trials. CRP, C‐reactive protein; Hb, haemoglobin; IGF‐1, insulin‐like growth factor 1; IL‐6, interleukin‐6; TNF, tumour necrosis factor.

*Figure* *2* summarizes the temporal trends in the most commonly studied biomarkers in cancer cachexia trials. Albumin has been used as a biomarker in cancer cachexia trials since at least 1993 and is still used today. As can be seen, readably obtainable biomarkers such as albumin, pre‐albumin, platelets, creatinine and haemoglobin have been in use since the 1990s. The use of more specific biomarkers, such as IGF‐1, has increased recently.

**Figure 3:**
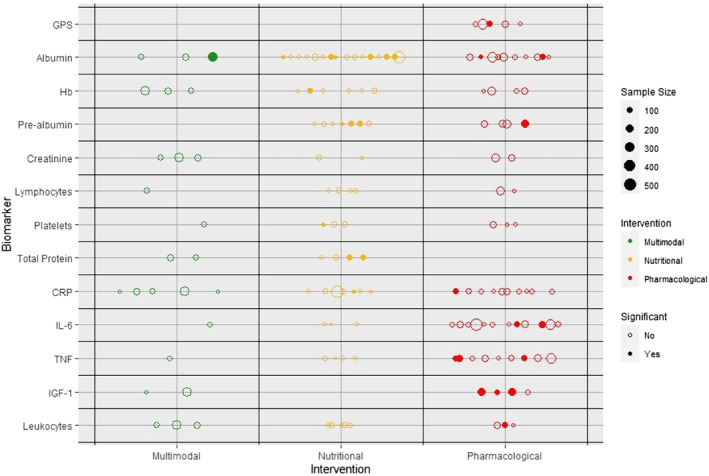
Balloon plot demonstrating the relationship between biomarkers and intervention in cancer cachexia trials. Trials yielding statistically significant changes between intervention arms are filled in. CRP, C‐reactive protein; GPS, Glasgow prognostic score; Hb, haemoglobin; IGF‐1, insulin‐like growth factor 1; IL‐6, interleukin‐6; TNF, tumour necrosis factor.

*Figure* *3* summarizes the 12 most commonly assessed biomarkers (used in six or more trials) and the most noteworthy biomarker, the Glasgow prognostic score (GPS). In total, 50 trials (96.2%) featured at least one of these 12 biomarkers. Albumin (*n* = 29, 55.8%; 3512 participants) was the most used biomarker in cancer cachexia trials. Nine of the 29 (31%) trials investigating albumin as a biomarker demonstrated statistically significant changes in serum levels between intervention arms. In five of these trials,^34^, ^35^, ^39^, ^60^, ^62^ albumin increased in the intervention arms, presumably as a result of less inflammation and/or improved nutritional status. In three studies, albumin decreased in both the intervention and control arms, but to a lesser extent in the intervention arms of two trials^20^, ^28^ and the control arm of another^29^ and one study. In the last of the nine studies with statistically significant changes reported in albumin, the data or direction were not presented.^57^ Six of these trials studied a nutritional intervention (*n* = 16, 37.5%), two studied a pharmacological intervention (*n* = 10, 20%) and one studied a multimodal intervention (*n* = 3, 66.6%).

The number of trials demonstrating a statistically significant change between intervention arms was comparable for albumin (9/29, 31%) and pre‐albumin (4/11, 36.4%). Following albumin, CRP was the next most observed biomarker (*n* = 22, 42.3%; 2481 participants). Two of these 22 trials (9.1%) showed statistically significant decreases in CRP levels between intervention arms, one in a nutritional intervention (*n* = 7, 14.3%) and one in a pharmacological intervention (*n* = 10, 10%).

The third most commonly used biomarker was interleukin (IL)‐6 (*n* = 16, 30.1%; 2101 participants). Statistically significant decreases in serum IL‐6 levels between intervention arms were observed only in two trials, and both studied a pharmacological intervention (2/12, 16.6%). Notably, among the 12 most used biomarkers, both creatinine (Cr) (0/7, 0%) and lymphocyte count (0/7, 0%) did not show any statistically significant changes in any trial.

Another biomarker of note was insulin‐like growth factor binding protein 3 (IGFBP‐3), which statistically significantly increased in all three trials in which it was studied. These three trials all studied anamorelin, a ghrelin analogue. The same three trials also observed statistically significant increases in IGF‐1. Nine other biomarkers had a significant change in the single trial in which they were featured. Six of these (docosapentaenoic acid [DPA], alpha lipoic acid [ALA], dihomo‐gamma‐linolenic acid [DGLA], gamma‐linolenic acid [GLA], linoleic acid [LA] and vitamin D) were studied across two trials,^24^, ^46^ which both featured a nutritional component to their intervention.

Pro‐inflammatory cytokines comprised 6 of the 99 biomarkers (6.1%). TNF‐α statistically significantly changed in the greatest number of studies (3/14, 12.5% [decreased in two trials^50^, ^52^ and increased in one trial^26^ but to a lesser extent than control]), followed by decreases in IL‐6 levels (2/16, *n* = 12.5%). As seen in *Figure* *3*, TNF‐α only changed significantly in studies with a pharmacological intervention, and this was the same for IL‐6. A low percentage of statistically significant change was also seen in the leucocyte count (decreased in 1/11 [9.1%]). IL‐8 (*n* = 2), IL‐15 (*n* = 1), IL‐1α (*n* = 1) and IL‐1β (*n* = 1) did not change significantly in any of the trials they featured in.

Three different biomarkers/biomarker scores were calculated from markers of inflammation: the GPS (*n* = 5), the neutrophil‐to‐lymphocyte ratio (*n* = 2) and the platelet‐to‐lymphocyte ratio (*n* = 1). Only GPS changed significantly between groups, and this was a decrease in one trial.^52^ All trials that included GPS used a pharmacological intervention.

## Discussion

This is the first systematic review to examine the use of biomarkers as endpoints in cancer cachexia. Many different biomarkers (*n* = 99) have been used across 52 trials, employing three different interventions (pharmacological, nutritional and multimodal) in at least seven different tumour types. The heterogeneity in trial design is evident, and biomarkers were used as a primary endpoint in six trials, with the remaining being an exploratory endpoint. Only one trial used a biomarker to guide power calculations, and 53.1% (*n* = 52) of biomarkers were only ever studied once. However, the landscape is shifting, and there is a trend towards the use of more specific biomarkers such as IGF‐1 and IL‐6. It is not possible to draw any robust conclusions about the many biomarkers presented in this review, as they are not featured frequently enough for patterns to emerge. A comment can be made on the more frequently assessed biomarkers, and in interpreting these findings, statistical significance or lack thereof does not necessarily mean that the biomarker is not useful. However, the effectiveness of the intervention, sample size calculations and sensitivity of the biomarker are all influencing factors.

Hypoalbuminaemia has long been recognized as a feature of cancer cachexia,^72^ and it is therefore expected that albumin was the most frequently used biomarker (29/52 trials), likely due to its dual role as a marker of inflammation and nutrition. Significant changes were only seen between trial arms in one third of the studies examining albumin. Due to the long half‐life of albumin (approximately 3 weeks), daily protein intake will have little immediate impact on serum levels,^73^ and consequentially, nutritional intervention only yielded 3 significant results of the 16 studies in which albumin was assessed following a nutritional intervention.

In pharmacological interventions, changes in albumin seemed to be dependent on the intervention. Studies that were positive used an anti‐inflammatory pharmacological intervention: indomethacin and thalidomide, which act to decrease levels of IL‐6^74^—albumin synthesis is inhibited by IL‐6,^75^ and this inflammatory mechanism is related to quality of life.^76^ These two studies suggest that when albumin is used as a biomarker in trials in which the inflammatory genesis of cachexia is targeted via an anti‐inflammatory intervention, it may prove valuable as an endpoint. In contrast, appetite stimulants increased oral intake but did not increase albumin.

With other biomarkers of the systemic inflammatory response, no substantial candidates were remarkable. Twenty‐two studies measured CRP as an endpoint, an acute‐phase protein with well‐documented prognostic value in cancer. Of note, in the two trials with statistically significant CRP results, both interventions included an anti‐inflammatory component. One studied indomethacin,^62^ and the other used a combination of eicosapentaenoic acid (EPA) and docosahexaenoic acid (DHA),^60^ which has been shown to have anti‐inflammatory properties.^77^ However, six other trials utilized a nonsteroidal anti‐inflammatory drug (NSAID) (pharmacological = four and multimodal = two) but did not show significant results. This is in keeping with a meta‐analysis of randomized controlled trials (RCTs) in patients with rheumatoid arthritis, which showed that NSAIDs have no effect on CRP levels.^78^ One of the pharmacological interventions used a drug that is not known to have any anti‐inflammatory properties (mirtazapine), suggesting that there is a disconnect between the biomarker chosen to be studied, the intervention and the cancer cachexia process. However, recently, the Global Leadership Initiative on Malnutrition consensus group has proposed that confirmation of inflammation should be guided by clinical judgement based upon the underlying diagnosis or condition, clinical signs or CRP.^79^ Furthermore, given that CRP and its derivatives, including albumin (mGPS and CRP‐to‐albumin ratio), are obligatory measurements in randomized clinical cancer trials,^80^ used extensively in immunotherapy trials,^81^, ^82^ and nutritional support will be increasingly given in this context, it is likely that CRP and its derivatives will become an increasingly important measurement in randomized cancer cachexia clinical trials.

When considering pro‐inflammatory cytokines, relatively few significant results were seen. A recent systematic review^83^ looked at the relationship between cytokines and symptoms in advanced cancer and found little correlation. This would suggest that trials need to focus more on measuring biomarkers involved in both the cachexia process and the intervention they have chosen to use. Other challenges with these include sampling errors, detection levels, paracrine/autocrine effects and sample timing.

This disconnect is less evident with regard to IGFBP‐3, IGF‐1 and anamorelin. Anamorelin is a ghrelin mimetic and growth hormone secretagogue that has been shown to increase levels of both IGFBP‐3 and IGF‐1.^84^ In all three studies, both IGFBP‐3 and IGF‐1 levels increased significantly in the intervention group. However, as mentioned previously, anamorelin has been shown to improve weight but not hand grip strength, and it is perhaps function that is arguably going to make the biggest improvement to the lives of people with cancer cachexia. If there is no clear correlation with a clinical benefit (e.g., does reducing CRP lead to increased function), then a biomarker will not fulfil FDA requirements to become a surrogate endpoint.

Overall, the reason behind the lack of trials showing significant improvements in biomarkers in cancer cachexia trials will be multifactorial. The first is a lack of efficacious treatment for cancer cachexia. The second is that many of the trials featured in this review were carried out across multiple tumour types. The underlying mechanisms in cancer cachexia are likely to be different in each cancer type^85^ (and perhaps each genotype), and therefore, applying one biomarker or set of biomarkers to test an intervention across multiple cancers is unlikely to show significant results; rather, patient selection may be based on the biomarker.

It is worth noting that in two recent narrative reviews of biomarkers in cancer cachexia,^5^, ^86^ both discussed using objective measures of the skeletal muscle wasting process, such as activin A and myostatin. They also suggested the use of GDF‐15 and parathyroid hormone‐related peptide (PTHrP). Neither of these biomarkers were featured in any of the trials in this review. The recent results of the first study of ponsegromab in participants with cancer cachexia^8^ are noteworthy due to it being the first cancer cachexia trial to admit patients based on the biomarker GDF‐15. However, given that this trial contained 10 participants, it did not meet the search criteria for this systematic review. The next phase of the study is eagerly anticipated.

### Strengths and limitations

The strengths of this review include its prospective design (PROSPERO) and use of broad search criteria encompassing the last three decades of cancer cachexia trials with data extraction performed by multiple independent reviewers. It is important to note the limitations of this review. In an effort to include clinical trials of a high standard, those with a smaller sample size (*n* < 40) were excluded; however, additional information could have been drawn from these trials and likewise from studies published before 1990. The use of the modified Downs and Black checklist provided a robust assessment in keeping with the other reviews in this endpoint series. Very few studies presented data on the association between the different biomarkers measured. Unless explicitly stated, it is often difficult to tease out whether authors considered outcomes as primary, secondary or exploratory. Therefore, only biomarkers that were explicitly stated as primary outcomes were highlighted in *Table* *1*, and this may underrepresent the perceived significance of some biomarkers. Again, unless explicitly stated, it is difficult to determine if routine biochemistry and haematology were measured in trials as endpoints or as part of the therapeutic monitoring process, and as such, the frequency of these biomarkers as outcomes is likely overstated.

A further limitation of the present review and studies herein was that the utility of a biomarker to predict those most likely to respond to an intervention and/or the reactiveness of a biomarker to the efficacy of an intervention was not assessed. Primarily, this was not only because the studies were rarely powered based on the biomarker being examined but also because the temporal relationship was not frequently described. This would be important to assess in future work, and if paradigms with oncology treatments were realized (e.g., PSA as both a diagnostic and treatment biomarker), this would be an important step for assessing who is most likely to benefit from a cachexia treatment and also to measure effectiveness.

## Conclusions

In conclusion, this systematic review of 52 cancer cachexia trials found 99 different biomarkers. Biomarkers are predominately used as exploratory rather than primary endpoints. It seems reasonable that for a biomarker to be responsive to change in the context of a cachexia clinical trial, it must be related to the mechanism of action of the intervention and/or the underlying cachexia process that is modified by the intervention. Further, to reach regulatory approval, the relationship between the biomarker and clinical benefit must be clear.

## Conflict of interest statement

SDA has received grants and personal fees from Vifor and Abbott Vascular and personal fees for consultancies, trial committee work and/or lectures from Actimed, Amgen, AstraZeneca, Bayer, Boehringer Ingelheim, BioVentrix, Brahms, Cardiac Dimensions, Cardior, Cordio, CVRx, Cytokinetics, Edwards, Farraday Pharmaceuticals, GSK, HeartKinetics, Impulse Dynamics, Novartis, Occlutech, Pfizer, Repairon, Sensible Medical, Servier, Vectorious and V‐Wave. He is the co‐inventor of two patent applications regarding MR‐proANP (DE 102007010834 and DE 102007022367), but he does not personally benefit from the related issued patents. JA has received personal fees from Danone. MF has received personal fees from Pfizer. MJH has received funding from CRUK, NIH National Cancer Institute, IASLC International Lung Cancer Foundation, Lung Cancer Research Foundation, Rosetrees Trust, UKI NETS and NIHR. MJH has consulted for and is a member of the Achilles Therapeutics Scientific Advisory Board and Steering Committee. MJH has received speaker honoraria from Pfizer, Astex Pharmaceuticals, Oslo Cancer Cluster and Bristol Myers Squibb and is a co‐inventor on a European patent application relating to methods to detect lung cancer (PCT/US2017/028013). BJAL has received personal fees for consulting from Artelo, Actimed, Faraday, Kyona Kirin and Toray. RJES has received personal fees for consulting from Artelo, Actimed, Faraday and Helsinn. EJR has a consulting/advisory role for Napo, AIM Specialty Health, Oragenics, BASF, Immuneering, Vector Oncology, Asahi Kasei, Heron, Pfizer/EMD Serono and Mitobridge.

## Supporting information


**Appendix S1.** Literature Search Strategy.

**Appendix S2.** Biomarkers.
