# Supplementary material for: Biomarker endpoints in cancer cachexia clinical trials: Systematic Review 5 of the cachexia endpoint series
Source: J Cachexia Sarcopenia Muscle. 2024 May 23;15(3):853–67. doi: 10.1002/jcsm.13491 (PMC11154797; doi:10.1002/jcsm.13491)
Supplement: Supplementary file 1 — Appendix S1. Literature Search Strategy. [file JCSM-15-853-s002.docx]

**Appendix 1: Literature Search Strategy**

1. Documentation on the literature search for “What is the optimal endpoint to evaluate effect of interventions aiming to treat cancer cachexia?”

The following databases were searched:

| **Database** | **Number of retrieved references for trials** | **Number of retrieved references for cohort/** **longitudinal studies** |
| --- | --- | --- |
| Medline (Ovid): | 3812 | 1918 |
| Embase (Ovid): | 2033 | 2031 |
| Cochrane Central Register of Controlled Trials: | 1923 |  |
| Number of references before de-duplication: | 8166 | 3949 |
| Number of references after de-duplication: | 5998 | 3190 |

All searches were done 02 June 2021 by Gunn Kleven, Senior librarian at the Library of Medicine and Science, University of Oslo. These were then updated to cover the period from June 2^nd^, 2023, until Oct., 17^th^ 2023, using the same search strategy as in the previous (until June 1^st^ 2023).

Number of hours spent: 34

**Ovid MEDLINE(R) ALL**1946 to October 17, 2023

Date searched: 02 June and updated 17 October 2023
Search Strategy:

| **#** | **Searches** | **Results** |
| --- | --- | --- |
| 1 | exp Neoplasms/ or (neoplasm* or cancer* or tumor* or tumour* or oncol* or malign* or carcinom* or adenocarcinom* or adenoma or metasta*).ti,ab,kf. | 4606655 |
| 2 | Cachexia/ or Emaciation/ or Malnutrition/ or Starvation/ or Wasting syndrome/ or Thinness/ or Sarcopenia/ or Anorexia/ or *Weight Loss/ | 63432 |
| 3 | and/1-2 | 9650 |
| 4 | ((cachexia or cachexic or anorexia or anorectic or emaciat* or malnutrition or underweight or starvation* or thiness or leanness or sarcopenia or wasting syndrome* or wasting disease* or weightloss* or ((appetite* or weight) adj2 (loss or loosing or losing))) adj4 (neoplasm* or cancer* or tumor* or tumour* or oncol* or malign* or carcinom* or adenocarcinom* or adenoma or metasta*)).ti,ab,kf. | 7231 |
| 5 | ((cachexia or cachexic or anorexia or anorectic or emaciat* or malnutrition or underweight or starvation* or thiness or leanness or sarcopenia or wasting syndrome* or wasting disease* or weightloss* or ((appetite* or weight) adj2 (loss or loosing or losing))) and (neoplasm* or cancer* or tumor* or tumour* or oncol* or malign* or carcinom* or adenocarcinom* or adenoma or metasta*)).ti. | 4253 |
| 6 | or/3-5 | 13924 |
| 7 | randomized controlled trial.pt. | 536354 |
| 8 | controlled clinical trial.pt. | 94265 |
| 9 | randomized.ab. | 525221 |
| 10 | placebo.ab. | 219320 |
| 11 | drug therapy.fs. | 2343029 |
| 12 | randomly.ab. | 360557 |
| 13 | trial.ab. | 557904 |
| 14 | groups.ab. | 2213680 |
| 15 | or/7-14 | 5047938 |
| 16 | exp animals/ not humans.sh. | 4855037 |
| 17 | 15 not 16 | 4388865 |
| 18 | 6 and 17 | 4078 |
| 19 | limit 18 to yr="1990 -Current" | 3812 |
| 20 | cohort studies/ or follow-up studies/ or longitudinal studies/ or "national longitudinal study of adolescent health"/ or prospective studies/ or retrospective studies/ | 2168707 |
| 21 | (cohort* or longitudinal or prospective* or retrospective*).tw. | 2098508 |
| 22 | or/20-21 | 3012965 |
| 23 | and/6,22 | 3215 |
| 24 | limit 23 to yr="1990 -Current" | 3139 |
| 25 | 24 not 19 | 1918 |
| 26 | 19 or 24 | 5730 |

**Embase Classic + Embase**1946 to October 17, 2023

Date searched: 02 June and updated 17 October 2023
Search Strategy:

| **#** | **Searches** | **Results** |
| --- | --- | --- |
| 1 | exp neoplasm/ or (neoplasm* or cancer* or tumor* or tumour* or oncol* or malign* or carcinom* or adenocarcinom* or adenoma or metasta*).ti,ab,kw. | 6400926 |
| 2 | cachexia/ or emaciation/ or *malnutrition/ or starvation/ or wasting syndrome/ or *anorexia/ or sarcopenia/ or *weight loss/ | 93608 |
| 3 | and/1-2 | 20654 |
| 4 | ((cachexia or cachexic or anorexia or anorectic or emaciat* or malnutrition or underweight or starvation* or thiness or leanness or sarcopenia or wasting syndrome* or wasting disease* or weightloss* or ((appetite* or weight) adj2 (loss or loosing or losing))) adj3 (neoplasm* or cancer* or tumor* or tumour* or oncol* or malign* or carcinom* or adenocarcinom* or adenoma or metasta*)).ti,ab,kw. | 9798 |
| 5 | ((cachexia or cachexic or anorexia or anorectic or emaciat* or malnutrition or underweight or starvation* or thiness or leanness or sarcopenia or wasting syndrome* or wasting disease* or weightloss* or ((appetite* or weight) adj2 (loss or loosing or losing))) and (neoplasm* or cancer* or tumor* or tumour* or oncol* or malign* or carcinom* or adenocarcinom* or adenoma or metasta*)).ti. | 6437 |
| 6 | or/3-5 | 24964 |
| 7 | Randomized controlled trial/ | 666248 |
| 8 | Controlled clinical trial/ | 463928 |
| 9 | random$.ti,ab. | 1691555 |
| 10 | randomization/ | 91413 |
| 11 | intermethod comparison/ | 272763 |
| 12 | placebo.ti,ab. | 330754 |
| 13 | (compare or compared or comparison).ti. | 571937 |
| 14 | ((evaluated or evaluate or evaluating or assessed or assess) and (compare or compared or comparing or comparison)).ab. | 2334019 |
| 15 | (open adj label).ti,ab. | 88467 |
| 16 | ((double or single or doubly or singly) adj (blind or blinded or blindly)).ti,ab. | 251401 |
| 17 | double blind procedure/ | 187924 |
| 18 | parallel group$1.ti,ab. | 27750 |
| 19 | (crossover or cross over).ti,ab. | 112863 |
| 20 | ((assign$ or match or matched or allocation) adj5 (alternate or group$1 or intervention$1 or patient$1 or subject$1 or participant$1)).ti,ab. | 359989 |
| 21 | (assigned or allocated).ti,ab. | 424479 |
| 22 | (controlled adj7 (study or design or trial)).ti,ab. | 385967 |
| 23 | (volunteer or volunteers).ti,ab. | 264717 |
| 24 | human experiment/ | 550005 |
| 25 | trial.ti. | 340818 |
| 26 | or/7-25 | 5516795 |
| 27 | (random$ adj sampl$ adj7 (cross section$ or questionnaire$1 or survey$ or database$1)).ti,ab. not (comparative study/ or controlled study/ or randomi?ed controlled.ti,ab. or randomly assigned.ti,ab.) | 8736 |
| 28 | Cross-sectional study/ not (randomized controlled trial/ or controlled clinical study/ or controlled study/ or randomi?ed controlled.ti,ab. or control group$1.ti,ab.) | 273554 |
| 29 | (((case adj control$) and random$) not randomi?ed controlled).ti,ab. | 18641 |
| 30 | (Systematic review not (trial or study)).ti. | 179350 |
| 31 | (nonrandom$ not random$).ti,ab. | 17184 |
| 32 | Random field$.ti,ab. | 2525 |
| 33 | (random cluster adj3 sampl$).ti,ab. | 1368 |
| 34 | (review.ab. and review.pt.) not trial.ti. | 902488 |
| 35 | we searched.ab. and (review.ti. or review.pt.) | 37332 |
| 36 | update review.ab. | 116 |
| 37 | (databases adj4 searched).ab. | 43700 |
| 38 | (rat or rats or mouse or mice or swine or porcine or murine or sheep or lambs or pigs or piglets or rabbit or rabbits or cat or cats or dog or dogs or cattle or bovine or monkey or monkeys or trout or marmoset$1).ti. and animal experiment/ | 1113420 |
| 39 | Animal experiment/ not (human experiment/ or human/) | 2339504 |
| 40 | or/27-39 | 3735626 |
| 41 | 26 not 40 | 4907360 |
| 42 | and/6,41 | 3904 |
| 43 | limit 42 to yr="1990 -Current" | 3674 |
| 44 | limit 43 to conference abstracts | 1641 |
| 45 | 43 not 44 | 2033 |
| 46 | cohort analysis/ or follow up/ or longitudinal study/ or "national longitudinal study of adolescent health"/ or prospective study/ or retrospective study/ | 3499550 |
| 47 | ((cohort adj (study or studies)) or cohort analy* or longitudinal).tw. | 705575 |
| 48 | or/46-47 | 3722608 |
| 49 | and/6,48 | 4417 |
| 50 | limit 49 to yr="1990 -Current" | 4387 |
| 51 | limit 50 to conference abstracts | 1639 |
| 52 | 50 not 51 | 2748 |
| 53 | 52 not 45 | 2031 |

**Cochrane Central Register of Controlled Trials:**

Date searched: 02 June and updated 17 October 2023

Search Strategy:

#1 [mh Neoplasms] 82548

#2 ((neoplasm* or cancer* or tumor* or tumour* or oncol* or malign* or carcinom* or

adenocarcinom* or adenoma or metasta*)):ti,ab,kw (Word variations have been searched) 232559

#3 #1 or #2 241300

#4 [mh Cachexia] or [mh ^Emaciation] or [mh ^Malnutrition] or [mh Starvation] or [mh

^"Wasting syndrome"] or [mh Thinness] or [mh Sarcopenia] or [mh Anorexia] 2665

#5 MeSH descriptor: [Weight Loss] this term only 6360

#6 #3 and (#4 or #5) 1017

#7 (((cachexia or cachexic or anorexia or anorectic or emaciat* or malnutrition or underweight

or starvation* or thiness or leanness or sarcopenia or "wasting syndrome" or "wasting syndromes" or "wasting disease" or "wasting diseases" or weightloss* or ((appetite* or weight) near/2 (loss or loosing or losing))) near/3 (neoplasm* or cancer* or tumor* or tumour* or oncol* or malign* or carcinom* or adenocarcinom* or adenoma or metasta*))):ti,ab,kw 1475

#8 (((cachexia or cachexic or anorexia or anorectic or emaciat* or malnutrition or underweight or starvation* or thiness or leanness or sarcopenia or "wasting syndrome" or "wasting syndromes" or "wasting disease" or "wasting diseases" or weightloss* or ((appetite* or weight) near/2 (loss or loosing or losing))) and (neoplasm* or cancer* or tumor* or tumour* or oncol* or malign* or carcinom* or adenocarcinom* or adenoma or metasta*))):ti (Word variations have been searched) 758

#9 #6 or #7 or #8 with Publication Year from 1990 to 2021, in Trials 2345
