# Supplementary material for: Biomarker endpoints in cancer cachexia clinical trials: Systematic Review 5 of the cachexia endpoint series
Source: J Cachexia Sarcopenia Muscle. 2024 May 23;15(3):853–67. doi: 10.1002/jcsm.13491 (PMC11154797; doi:10.1002/jcsm.13491)
Supplement: Supplementary file 2 — Appendix S2. Biomarkers. [file JCSM-15-853-s001.docx]

**Appendix 2: Biomarkers**

| **Biomarker** | **Number** | **Significant** | **%** |
| --- | --- | --- | --- |
| Albumin | 29 | 9 | 31.0% |
| CRP | 22 | 2 | 9.1% |
| Interleukin 6 (IL-6) | 16 | 2 | 12.5% |
| Tumour Necrosis Factor Alpha (TNF- α) | 14 | 3 | 21.4% |
| Haemoglobin | 13 | 1 | 7.7% |
| Leucocytes | 11 | 1 | 9.1% |
| Pre-Albumin | 11 | 4 | 36.4% |
| Creatinine | 7 | 0 | 0.0% |
| Lymphocytes | 7 | 0 | 0.0% |
| Platelets | 7 | 1 | 14.2% |
| Insulin-like Growth Factor (IGF-1) | 6 | 3 | 50.0% |
| Total Protein | 6 | 2 | 33.3% |
| Alanine Transaminase (ALT) | 5 | 0 | 0.0% |
| Aspartate Transaminase (AST) | 5 | 0 | 0.0% |
| Glasgow Prognostic Score (GPS) | 5 | 1 | 20.0% |
| Haematocrit | 5 | 1 | 20.0% |
| Transferrin | 5 | 1 | 20.0% |
| Triglycerides | 5 | 0 | 0.0% |
| Eicosapentaenoic acid (EPA) | 4 | 2 | 50.0% |
| Glucose | 4 | 2 | 50.0% |
| Thrombocytes | 4 | 0 | 0.0% |
| Total cholesterol | 4 | 0 | 0.0% |
| Docosahexaenoic Acid (DHA) | 3 | 1 | 33.3% |
| Iron | 3 | 1 | 33.3% |
| Ferritin | 3 | 1 | 33.3% |
| HbA1c | 3 | 0 | 0.0% |
| High-Density Lipoprotein (HDL) | 3 | 0 | 0.0% |
| Insulin-like Growth Factor Binding Protein (IGFBP-3) | 3 | 3 | 100.0% |
| Low Density Lipoprotein (LDL) | 3 | 0 | 0.0% |
| Neutrophils | 3 | 0 | 0.0% |
| Urea | 3 | 2 | 66.6% |
| Alkaline Phosphatase (ALP) | 2 | 1 | 50.0% |
| Arachidonic acid | 2 | 0 | 0.0% |
| Bilirubin | 2 | 0 | 0.0% |
| Cholesterol | 2 | 0 | 0.0% |
| Cholinesterase | 2 | 0 | 0.0% |
| Erythrocyte Sedimentation Rate (ESR) | 2 | 1 | 50.0% |
| Free T3 | 2 | 1 | 50.0% |
| Glutathione Peroxidase | 2 | 0 | 0.0% |
| Interleukin 8 (IL-8) | 2 | 0 | 0.0% |
| Insulin | 2 | 1 | 50.0% |
| Mean Corpuscular Volume (MCV) | 2 | 0 | 0.0% |
| Neutrophil: Lymphocyte ratio | 2 | 0 | 0.0% |
| Prostate Specific Antigen (PSA) | 2 | 0 | 0.0% |
| Reactive Oxygen Species (ROS) | 2 | 1 | 50.0% |
| Urinary Nitrogen | 2 | 0 | 0.0% |
| Acylghrelin | 1 | 0 | 0.0% |
| Adiponectin | 1 | 0 | 0.0% |
| Bone-Specific Alkaline Phosphatase | 1 | 0 | 0.0% |
| CA19-9 | 1 | 0 | 0.0% |
| CD14 expression | 1 | 0 | 0.0% |
| CD16 expression | 1 | 0 | 0.0% |
| CD4+ | 1 | 0 | 0.0% |
| Ceruloplasmin | 1 | 0 | 0.0% |
| Cobalamin | 1 | 0 | 0.0% |
| C-telopeptide | 1 | 0 | 0.0% |
| Desacylghrelin | 1 | 0 | 0.0% |
| Docosapentaenoic Acid (DPA) | 1 | 1 | 100.0% |
| Folate | 1 | 0 | 0.0% |
| Free testosterone | 1 | 0 | 0.0% |
| Follicular Stimulating Hormone (FSH) | 1 | 0 | 0.0% |
| Ghrelin | 1 | 0 | 0.0% |
| Glycerol | 1 | 0 | 0.0% |
| Glutathione (GSH) | 1 | 1 | 100.0% |
| Haptoglobin | 1 | 0 | 0.0% |
| IgG | 1 | 1 | 100.0% |
| Interleukin 15 (IL-15) | 1 | 0 | 0.0% |
| Interleukin 1α (IL-1α) expression | 1 | 0 | 0.0% |
| Interleukin 1β (IL-1β) | 1 | 0 | 0.0% |
| LDL/HDL ratio | 1 | 0 | 0.0% |
| Leptin | 1 | 1 | 100.0% |
| Luteinizing Hormone (LH) | 1 | 0 | 0.0% |
| Lipolytic activity | 1 | 0 | 0.0% |
| Magnesium | 1 | 0 | 0.0% |
| Mean Corpuscular Haemoglobin (MCH) | 1 | 0 | 0.0% |
| Alpha-linolenic Acid (ALA) | 1 | 1 | 100.0% |
| Dihomo-gamma-linolenic Acid (DGLA) | 1 | 1 | 100.0% |
| Gamma-linolenic acid (GLA) | 1 | 1 | 100.0% |
| Linoleic Acid (LA) | 1 | 1 | 100.0% |
| N-telopeptide | 1 | 0 | 0.0% |
| Osteocalcin | 1 | 0 | 0.0% |
| Phosphate | 1 | 0 | 0.0% |
| Phospholipids | 1 | 0 | 0.0% |
| Platelet: Lymphocyte ratio | 1 | 0 | 0.0% |
| Reticulocytes | 1 | 0 | 0.0% |
| Red Blood Cells | 1 | 0 | 0.0% |
| Red Cell Distribution Width (RDW) | 1 | 0 | 0.0% |
| Retinol Binding Protein | 1 | 0 | 0.0% |
| Superoxide Dismutase | 1 | 0 | 0.0% |
| Thyroxine Binding Prealbumin (TBPA) | 1 | 0 | 0.0% |
| Total Iron Binding Capacity | 1 | 0 | 0.0% |
| Total Testosterone | 1 | 0 | 0.0% |
| Urinary Creatinine | 1 | 0 | 0.0% |
| Very Low-Density Lipoprotein (VLDL) | 1 | 0 | 0.0% |
| Vitamin D | 1 | 1 | 100.0% |
| YKL-40 | 1 | 0 | 0.0% |
| Zinc-α2-glycoprotein | 1 | 0 | 0.0% |
| α-1 antitrypsin | 1 | 0 | 0.0% |
| α-1-acid glycoprotein | 1 | 0 | 0.0% |
